# Significantly Enhanced Dielectric Properties of Ag-Deposited (In_1/2_Nb_1/2_)_0.1_Ti_0.9_O_2_/PVDF Polymer Composites

**DOI:** 10.3390/polym13111788

**Published:** 2021-05-28

**Authors:** Wattana Tuichai, Pornsawan Kum-onsa, Supamas Danwittayakul, Jedsada Manyam, Viyada Harnchana, Prasit Thongbai, Nutthakritta Phromviyo, Prinya Chindaprasirt

**Affiliations:** 1 Giant Dielectric and Computational Design Research Group (GD–CDR), Department of Physics, Faculty of Science, Khon Kaen University, Khon Kaen 40002, Thailand; champy_akat@hotmail.com (W.T.); pornsawan.kumonsa@gmail.com (P.K.-o.); viyada@kku.ac.th (V.H.); 2 National Metal and Materials Technology Center, National Science and Technology Development Agency, Thailand Science Park, Pathumthani 12120, Thailand; supamasd@mtec.or.th; 3 National Nanotechnology Center (NANOTEC), National Science and Technology Development Agency (NSTDA), Pathum Thani 12120, Thailand; jedsada@nanotec.or.th; 4 Institute of Nanomaterials Research and Innovation for Energy (IN–RIE), NANOTEC–KKU RNN on Nanomaterials Research and Innovation for Energy, Khon Kaen University, Khon Kaen 40002, Thailand; 5 Sustainable Infrastructure Research and Development Center, Department of Civil Engineering, Faculty of Engineering, Khon Kaen University, Khon Kaen 40002, Thailand; nutthaphrom@gmail.com (N.P.); prinya@kku.ac.th (P.C.)

**Keywords:** silver nanoparticles, colossal permittivity, TiO_2_, polyvinylidene fluoride (PVDF), polymeric composite, hybrid particle

## Abstract

The enhanced dielectric permittivity (ε′) while retaining a low loss tangent (tanδ) in silver nanoparticle−(In_1/2_Nb_1/2_)_0.1_Ti_0.9_O_2_/poly(vinylidene fluoride) (Ag-INTO/PVDF) composites with different volume fractions of a filler (*f*_Ag-INTO_) was investigated. The hybrid particles were fabricated by coating Ag nanoparticles onto the surface of INTO particles, as confirmed by X-ray diffraction. The ε′ of the Ag−INTO/PVDF composites could be significantly enhanced to ~86 at 1 kHz with a low tanδ of ~0.044. The enhanced ε′ value was approximately >8-fold higher than that of the pure PVDF polymer for the composite with *f*_Ag-INTO_ = 0.5. Furthermore, ε′ was nearly independent of frequency in the range of 10^2^–10^6^ Hz. Therefore, filling Ag−INTO hybrid particles into a PVDF matrix is an effective way to increase ε′ while retaining a low tanδ of polymer composites. The effective medium percolation theory model can be used to fit the experimental ε′ values with various *f*_Ag-INTO_ values. The greatly increased ε′ primarily originated from interfacial polarization at the conducting Ag nanoparticle–PVDF and Ag–INTO interfaces, and it was partially contributed by the high ε′ of INTO particles. A low tanδ was obtained because the formation of the conducting network in the polymer was inhibited by preventing the direct contact of Ag nanoparticles.

## 1. Introduction

A new method to enhance the dielectric permittivity (ε′) of polymer matrix composites was proposed to obtain high-performance dielectric properties of polymer-based materials for use in capacitors, resistors, and embedded devices [1,2,3,4]. Many research groups have focused on the development of polymer nanocomposites with high ε′ and low loss tangent (tanδ), which can be considered as candidate dielectric polymers [1,2,4,5,6].

A simple method for achieving an improved dielectric response in polymers is to fill high-ε′ oxides, e.g., and ACu_3_Ti_4_O_12_ (ACTO) (A = Ca, Na_1/2_Bi_1/2_, Na_1/2_Y_1/2_, and Na_1/3_Ca_1/3_Bi_1/3_) [7,8,9,10,11], La_2-*x*_Sr*_x_*NiO_4_ (LSNO) [5,6,12], TiO_2_ nanowires [13], K_0.5_Na_0.5_NbO_3_-SrTiO_3_ [14], and BaTiO_3_ (BT) [15,16,17,18,19], into the polymer. The enhanced dielectric properties are attributed to the high ε′ of the filler and interfacial polarization at the interface of the polymer matrix and filler particles. Although the ε′ value of ceramic−polymer composites can be increased with increasing volume fractions of a filler (*f*_filler_), the highly deteriorated dissipation factor of energy (tanδ >> 1) limits their application as dielectric materials. This is due to the high tanδ of ceramic fillers used. In some composite systems, the ε′ value is difficult to enhance even when *f*_filler_ ≥ 0.5.

Another simple method to enhance the ε′ of dielectric polymers is to incorporate conductive nanoparticles or nanofibers (e.g., Ag [20,21], Ni [22], Al [23], or multiwalled carbon nanotubes [24]) into the polymer matrix at *f*_filler_ close to the percolation threshold (*f*_c_). In particular, Ag is attractive due to its high conductivity, low cost, and ease of synthesis. Unfortunately, an enhanced ε′ value of metal–polymer composites is usually accompanied by a large leakage current (*I*_L_) and high tanδ when *f*_filler_ → *f*_c_. The increases in *I*_L_ and tanδ are unsuitable for applications of polymer composites.

In recent years, an effective route to improve the dielectric properties of polymer−matrix composites has been to use hybrid fillers consisting of ceramic particles deposited by metal nanoparticles such as Ag–TiO_2_ [25,26], Ag–La_1.9_Sr_0.1_Ni_0.6_Mg_0.4_O_4_ [27], Au–BT [3], Ag–BT [28,29], Au–ACTO [30], Au–BiFeO_3_ [31], and Ag–ACTO [32,33]. The improved dielectric properties of the polymer composites filled with these hybrid particles are caused by intensive interfacial metal–ceramic, metal–polymer, and ceramic–polymer interactions. The enhanced conductivity and tanδ due to incorporation with conductive particles can be inhibited by the discrete growth of metal particles on the surface of ceramic particles. Many previous works showed that a significantly enhanced ε′ and low tanδ of these three-phase composites are achieved [3,26,30,31,32,33,34,35].

Hu et al. [36] reported a new class of giant dielectric material, i.e., In^3+^/Nb^5+^ co-doped rutile-TiO_2_. When the co-doping concentration was 10%, a low tanδ of ~0.02 and high ε′ ~6 × 10^4^ were achieved over a frequency range of 10^2^−10^6^ Hz. Notably, the ε′ was slightly dependent on temperature in the temperature range of 80−450 K. The observed dielectric behavior was explained by the formation of complex defect clusters inside the grains due to the substitution of aliovalent dopants. The overall dielectric properties of the In^3+^/Nb^5+^ co-doped TiO_2_ ceramics are better than those of conventional giant dielectric oxides.

Considering a conventional ceramic/PVDF composite system, the observed high tanδ value of the ceramic/PVDF composites likely results from high values of the conductivity and tanδ of the ceramic filler used, especially for ACTO/PVDF, LSNO/PVDF, and Ba(Fe_0.5_Nb_0.5_)O_3_/PVDF composites [6,7,8,37]. For these ceramics, the obtained high ε′ of ~10^4^ is caused by the extrinsic effect associated with their semiconducting grains and internal insulating layer. These ceramic particles can be considered as semiconducting fillers in a polymer matrix composite because there is no internal insulating layer (e.g., grain boundary) in filler particles used. In contrast, although a low tanδ value can be achieved in a conventional BT/PVDF composite system, a significantly enhanced ε′ response is difficult to achieve due to a low intrinsic ε′ value associated with the ferroelectricity and the absence of strong interfacial polarization. Therefore, all of these ceramic particles may be an improper choice for use as a filler in a two-phase ceramic/polymer composite or unsuitable for fabricating a hybrid particle.

Instead, In^3+^/Nb^5+^ co-doped TiO_2_ particles may be an attractive choice because the obtained high ε′ and low tanδ values are associated with the quasi-intrinsic effect. The macroscopic point of view of the In^3+^/Nb^5+^ co-doped TiO_2_ particles involves insulating particles with very high resistivity compared to that of other giant dielectric oxides. Nevertheless, the INTO particles contain highly localized free electrons in the clusters of defects, giving rise to the significantly increased ε′. Thus, the quasi-intrinsic ε′ value of the INTO particles is expected to be much higher than that of the intrinsic ε′ value of the BT particles. Tes et al. [38] reported the significantly enhanced dielectric properties of modified surface (Er_0.5_ + Nb_0.5_)*_x_*Ti_1−*x*_O_2_/P(VD–-TrFE) polymer composites. Furthermore, it was reported that the (La, Nb) co-doped TiO_2_/silicone rubber composites can exhibit greatly enhanced dielectric properties [39].

By using a hybrid particle approach for improving the dielectric properties of polymer composites, Ag-(In^3+^/Nb^5+^) co-doped TiO_2_ hybrid particles may, therefore, be one of the most exciting choices. If the origin of the giant dielectric response in the In^3+^/Nb^5+^ co-doped TiO_2_ ceramics is attributed to the quasi-intrinsic effect, a significantly increased ε′ value while retaining low tanδ should be achieved in the composites. Poly(vinylidene fluoride) (PVDF) is one of the most significant dielectric polymers owing to its high ε′ ~10−12 compared to that of other polymers [1]. Thus, the objective of this work was to enhance the dielectric properties of PVDF polymer composites by incorporating Ag-(In^3+^/Nb^5+^) co-doped TiO_2_ hybrid particles.

In this study, we fabricated a new composite system consisting of Ag-(In^3+^/Nb^5+^) co-doped TiO_2_ hybrid particles incorporated in a PVDF polymer matrix to obtain a largely enhanced ε′ with low tanδ. The effect of filler volume fraction on the variation in dielectric properties was investigated. The microstructure, morphology, and dielectric properties were discussed in detail to possibly justify whether such composites are designable for applications in next-generation electronics.

## 2. Experimental Details

### 2.1. Preparation of (In_1/2_Nb_1/2_)_0.1_Ti_0.9_O_2_ powders

The (In_1/2_Nb_1/2_)_0.1_Ti_0.9_O_2_ (INTO) powder was prepared by a conventional mixed-oxide method. Firstly, In_2_O_3_ (Sigma−Aldrich, Saint Louis, MO, USA, 99.9% purity), Nb_2_O_5_ (Sigma−Aldrich, Saint Louis, MO, USA, 99.99% purity), and rutile-TiO_2_ (Sigma−Aldrich, Saint Louis, MO, USA, >99.9% purity) were mixed using a wet ball-milling method in ethanol for 24 h. Secondly, ethanol was evaporated at 80 °C for 24 h. Lastly, the mixed dried powder was calcined at 1100 °C for 10 h to obtain an INTO powder.

### 2.2. Preparation of Ag−INTO Hybrid Particles

Ag−INTO hybrid particles were prepared using a seed-mediated growing process by a redox reaction between silver nitrate and ethylene glycol. Firstly, AgNO_3_ (RCI Labscan, Bangkok, Thailand, 99.8% purity) was dissolved in 300 mL of ethylene glycol. Secondly, INTO was added to ethylene glycol under constant magnetic stirring for 2 h at ~30 °C. Then, the temperature of the mixed solution was increased to 140 °C for 25 min. Next, the Ag−INTO powders in ethylene glycol were washed with absolute ethanol three times. Lastly, the Ag−INTO hybrid particles were obtained by heating in an oven at 80 °C for 1 h.

### 2.3. Preparation of Ag−INTO/PVDF Nanocomposites

Ag−INTO/PVDF composites with different volume fractions of Ag−INTO hybrid particles (*f*_Ag__−INTO_ = 0–0.55) were prepared by a liquid-phase-assisted dispersion method [1]. Firstly, Ag−INTO and PVDF particles were mixed by a ball-milling method in absolute ethanol for 30 min using ZrO_2_ balls. Secondly, the mixture of Ag−INTO and PVDF particles was dried to evaporate absolute ethanol at 80 °C. Lastly, the mixed powders were molded by hot-pressing at 200 °C for 30 min. The final disc shape of the composite samples was 12 mm in diameter and 1 mm in thickness.

### 2.4. Characterization Techniques and Dielectric Measurement

X-ray diffraction (XRD; PANalytical, Malvern, UK, EMPYREAN) was used to characterize the phase composition of the composite samples. The morphologies of the Ag−INTO hybrid particles and Ag−INTO/PVDF were revealed by transmission electron microscopy (TEM; FEI Tecnai G2 20) and scanning electron microscopy (SEM) (SEC, SNE-4500M), respectively. Before dielectric measurements, the disc samples were painted with silver paste on both sides of the surface. Then, the electrode was dried at 150 ℃ for 0.5 h. The capacitance (*C*_p_) and dissipation factor (tan*δ*) of the Ag−INTO/PVDF composites were measured using an impedance analyzer (KEYSIGHT, E4990A) under an AC oscillation voltage of 0.5 V over a frequency range of 10^2^−10^6^ Hz and a temperature range of −60 to 150 °C.

## 3. Results and Discussion

**Figure 1:**
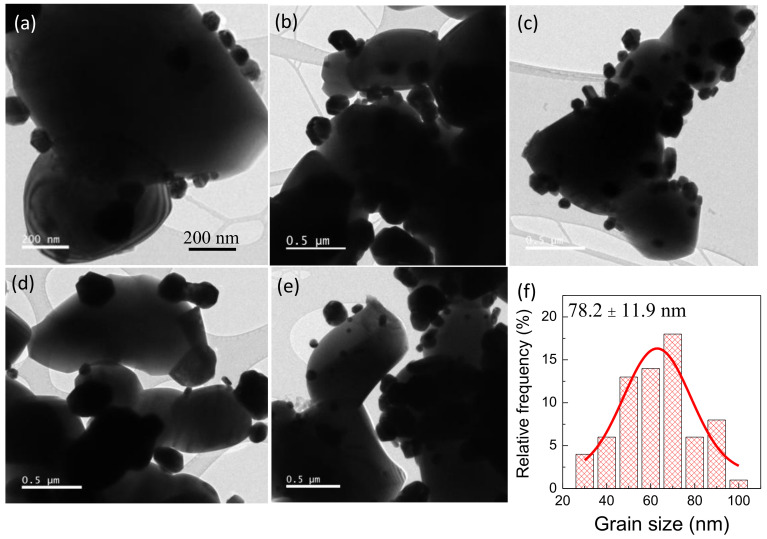
(**a**–**e**) TEM images of Ag–INTO hybrid particles prepared by a seed-mediated growing process; (**f**) size distribution of Ag–INTO particles.

The surface morphology of Ag−INTO hybrid particles was revealed using the TEM technique. As shown in Figure 1a–e, the formation of of Ag−INTO hybrid particles was confirmed. The Ag particles had a nearly spherical shape, while the INTO particles had an irregular shape with sizes ranging from ~1–2 μm. The surface of INTO could be discretely deposited with discrete Ag nanoparticles, forming the Ag−INTO hybrid particles for use as a filler in the PVDF polymer matrix. The particle size of Ag nanoparticles was similar to that reported in the literature [35]. Figure 1f shows the size distribution of the Ag nanoparticles. Accordingly, the average particle size of the Ag nanoparticles was 78.2 ± 11.9 nm.

**Figure 2:**
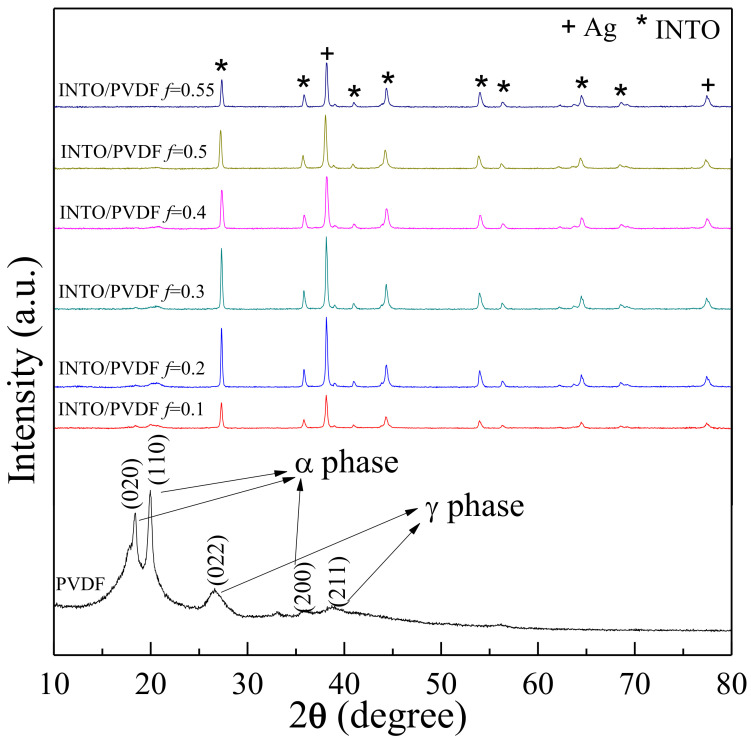
XRD patterns of pure PVDF and Ag−INTO/PVDF composites with various *f*_Ag__−INTO_ = 0−0.55.

The phase formation of pure PVDF and Ag−INTO/PVDF was investigated, as shown in Figure 2. The XRD result indicates the formation of α- and γ-PVDF phases [7,8]. It was confirmed that the prepared Ag−INTO/PVDF composites consisted of a rutile-TiO_2_ phase (JCPDS 21-1276) without any possible impurity of Nb- or In-related phase and Ag phase (JCPDS 89–3722). The XRD pattern of the PVDF phase in the Ag−INTO/PVDF composites decreased with increasing *f*_Ag__−INTO_ due to a high crystallinity of the Ag and INTO phases compared to that of the semicrystalline PVDF polymer. According to the XRD pattern of the Ag−INTO hybrid particles (not shown), the weight ratio of INTO and Ag was calculated to be 76.2:23.8, respectively.

**Figure 3:**
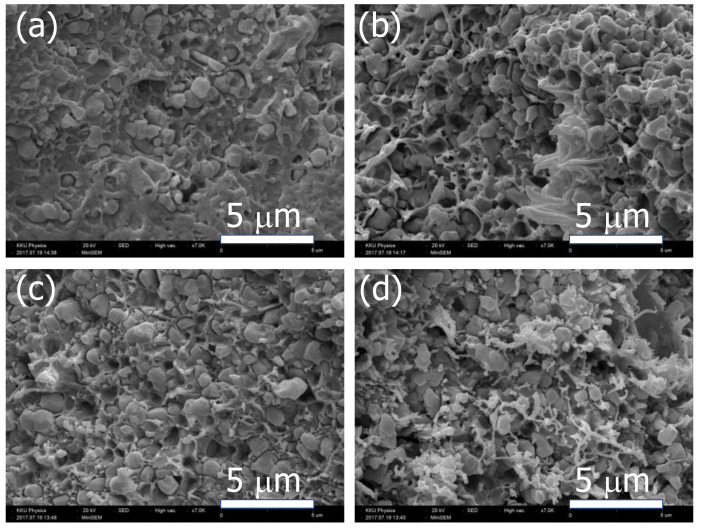
SEM images of Ag−INTO/PVDF composites with different *f*_Ag__−INTO_ values: (**a**) *f*_Ag__−INTO_ = 0.1, (**b**) *f*_Ag__−INTO_ = 0.3, (**c**) *f*_Ag__−INTO_ = 0.4, and (**d**) *f*_Ag__−INTO_ = 0.5.

Figure 3a–c show the SEM images of the fractured cross-section of the Ag−INTO/PVDF composites with *f*_Ag__−INTO_ = 0.1, 0.3, and 0.5, respectively, which were prepared by a liquid-phase-assisted dispersion method. For the composite with *f*_Ag__−INTO_ = 0.1, it was observed that the Ag−INTO hybrid particles randomly dispersed throughout the PVDF polymer matrix with sizes of ~1–2 μm. A highly dense microstructure was achieved. For the composite with *f*_Ag__−INTO_ = 0.3−0.5, the interparticle distance between the Ag−INTO hybrid particles was reduced by increasing filler concentration. Generally, the ε′ and tanδ of the composites are dependent on the dispersion of filler and interparticle distance [6]. Furthermore, a greater degree of aggregation and some pores were observed in the Ag−INTO/PVDF composites as the *f*_Ag__−INTO_ increased. A continuous phase of PVDF polymer can be observed in all the composites.

**Figure 4:**
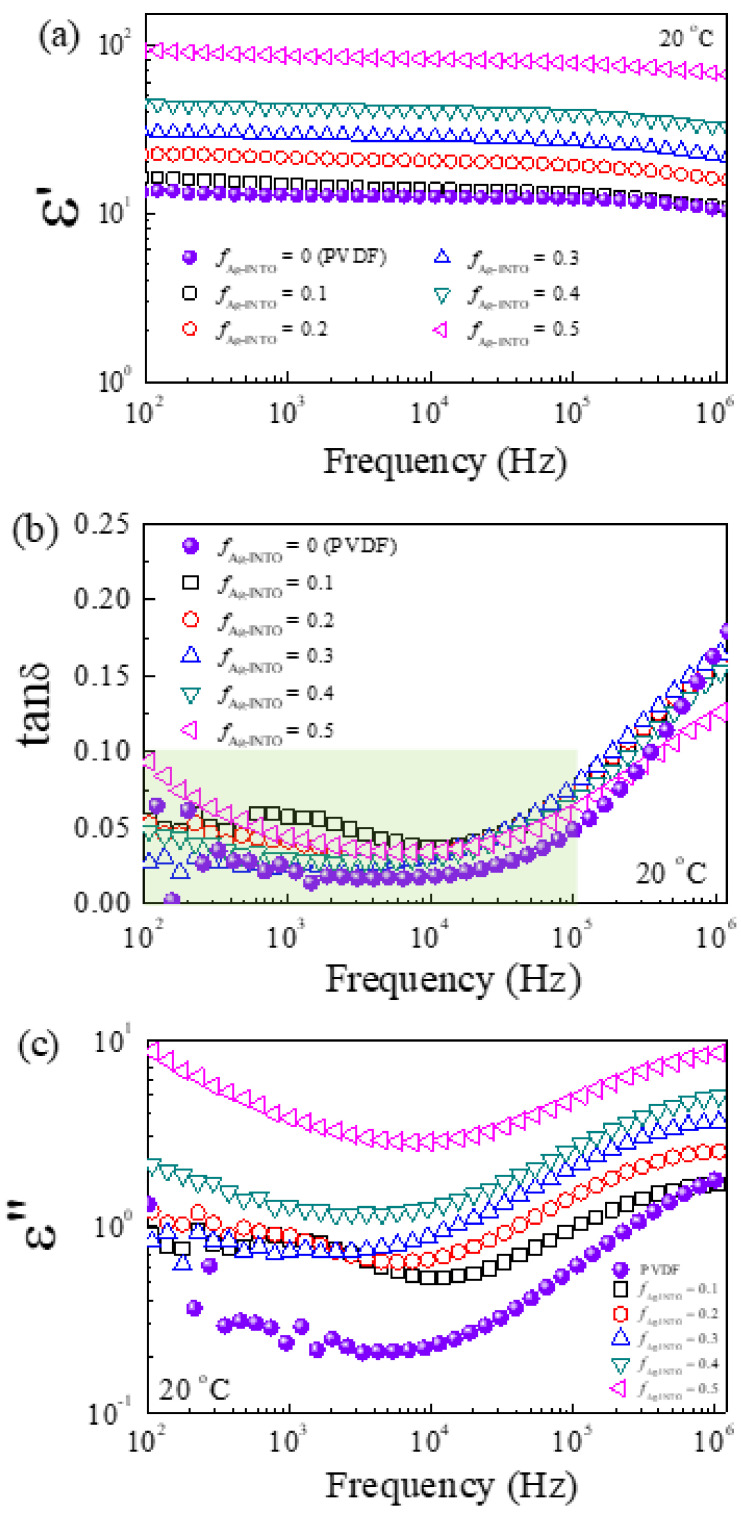
Frequency dependence of (**a**) ε′, (**b**) tanδ, and (**c**) ε″ at 20 °C for PVDF polymer and Ag−INTO/PVDF composites with different *f*_Ag__−INTO_ values.

Figure 4 shows the dielectric parameters (ε′ and tanδ) at 20 °C as a function of frequency for the Ag−INTO/PVDF composites with various *f*_Ag__−INTO_ values. Figure 4a shows that the ε′ at 20 °C of the composites was nearly independent of the frequency ranging from 10^2^ to 10^6^ Hz. A slight decrease in ε′ was observed in the frequency range from 10^5^ to 10^6^ Hz, corresponding to a sharp increase in tanδ, as shown in Figure 4b. This dielectric behavior in a high-frequency range is a Debye relaxation behavior. The relaxation is caused by the C−F dipole orientation polarization of the PVDF matrix [37]. On the other hand, the low-frequency tanδ was likely due to the interfacial polarization effect. The ε′ was significantly enhanced as the *f*_Ag__−INTO_ values increased. Notably, significantly improved dielectric properties of the Ag−INTO/PVDF composite with various *f*_Ag__−INTO_ = 0.5 were achieved. The ε′ values at low frequencies were as high as ~92.2 and ~85.9 at 10^2^ and 10^3^ Hz, respectively. These values were approximately nine- and eightfold higher than those of the PVDF polymer at 10^2^ and 10^3^ Hz, respectively. The significantly increased ε′ of the Ag−INTO/PVDF composites was likely related to the high ε′ of the INTO particles and the incorporation of Ag nanoparticles. This is comprehensively discussed in the last part of this section using a theoretical model. Maxwell−Wagner–Sillars (MWS) polarization [40], which is usually the primary cause for the significant increase in ε′ of the (semi)conductor−insulator composites, may likely exist in the internal interfaces, i.e., Ag−INTO and Ag−PVDF interfaces. MWS polarization resulted from the accumulated free charges in the discontinuous surfaces. Under an electric field, this led to the induced macroscopic dipoles, giving rise to the enhancement of the effective ε′ of the composites.

The tanδ of Ag−INTO/PVDF composites remained low at frequencies below 10^5^ Hz (Figure 4b), which is lower than that of many two-phase composite systems [7,10,37]. The three-phase composite with *f*_Ag__−INTO_ = 0.5 had a tanδ of 0.044 at 1 kHz. The suppressed tanδ value of the Ag−INTO/PVDF composites was associated with the characteristic of the modified surface of Ag−INTO hybrid particles, which can still be considered an insulating interface because the direct contact between Ag nanoparticles was inhibited by the discrete growth of Ag nanoparticles, preventing leakage current and, hence, tanδ [41]. A slight increase in tanδ at low frequencies of the composite with *f*_Ag__−INTO_ = 0.5 was due to the interfacial polarization effect owing to the relaxation loss caused by MWS interfacial polarization [42,43]. Figure 4c shows the frequency dependence of the dielectric loss (ε′′ = ε′ × tanδ). As clearly seen, the increased ε′′ in a low-frequency range confirms the existence of interfacial polarization. Moreover, the sharp increases in both tanδ and ε′′ in the high-frequency range of 10^5^−10^6^ Hz originated from α_a_ relaxation due to C−F dipole orientation polarization [42,43].

**Figure 5:**
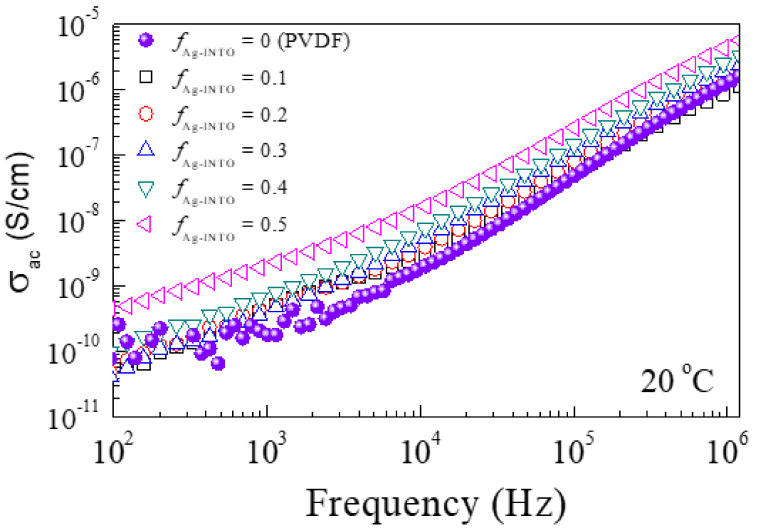
Frequency dependence of σ_ac_ at 20 °C for PVDF polymer and Ag−INTO/PVDF composites with different *f*_Ag__−INTO_ values.

The frequency dependence of AC conductivity (σ_ac_) of Ag−INTO/PVDF composites is shown in Figure 5. Over the measured frequency range, when the filler concentration of the hybrid particles was increased, σ_ac_ was increased slightly. At *f*_Ag__−INTO_ = 0.5, the σ_ac_ value at 1 kHz was only 2.13 × 10^−9^ S/cm at 1 kHz. This is much lower than that of other three-phase composite systems, which is usually >10^−7^ S/cm [25,34]. Thus, it is clearly confirmed that no conducting pathway was formed. The composites showed good insulation properties even when the PVDF polymer was filled with conductive Ag nanoparticles.

**Figure 6:**
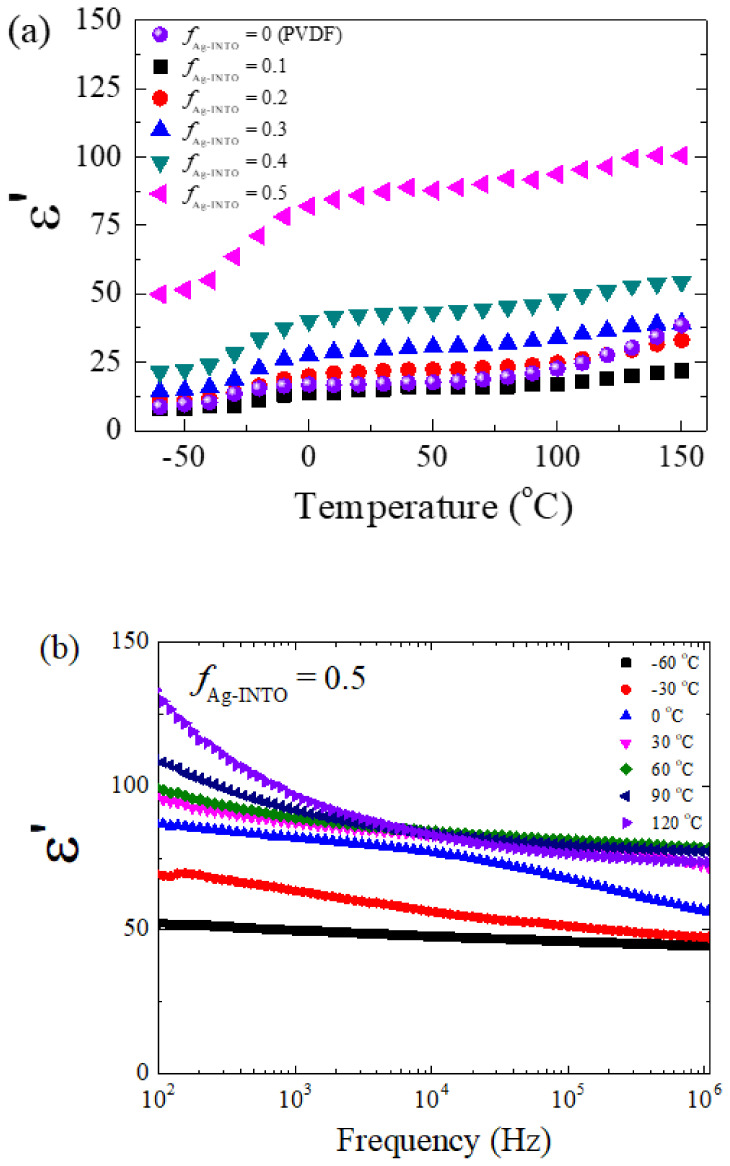
(**a**) Temperature dependence of ε′ at 1 kHz for PVDF polymer and Ag−INTO/PVDF composites with different *f*_Ag__−INTO_ values. (**b**) Frequency dependence of ε′ at different temperatures for Ag−INTO/PVDF composite with *f*_Ag__−INTO_ = 0.5.

The ε′ as a function of temperature at 1 kHz of the Ag−INTO/PVDF composites with various *f*_Ag__−INTO_ values was studied. As shown in Figure 6a, the ε′ of the Ag−INTO/PVDF composites showed good thermal stability in the range of 60−150 ℃. With increasing temperature, the ε′ values of all samples increased slightly. A decrease in ε′ at low temperatures resulted from kinetics related to the freezing of the dipole moments [44]. This result was described as a relaxation of the polar phase in the PVDF matrix, which usually appears around −35 ℃ [45]. As shown in Figure 4 and Figure 6, significantly enhanced dielectric properties of the Ag−INTO/PVDF composites with *f*_Ag__−INTO_ = 0.05 were obtained. The low tanδ and high ε′ with good temperature stability of the ε′ are suitable for applications in capacitors.

The frequency dependence of ε′ at different temperatures for the composite with *f*_Ag__−INTO_ = 0.05 is illustrated in Figure 6b. At −60 °C, the dielectric data show the plateau with ε′ ~50 over the measured frequency range. The increased ε′ value from ~10 for the PVDF polymer matrix to ~50 for the composite with *f*_Ag__−INTO_ = 0.05 was likely contributed by the polarization in the INTO particles. At such a low temperature, the dielectric response of the interfacial polarization at the Ag–INTO and Ag–PVDF interfaces could not be induced even at the lowest frequency of 10^2^ Hz. The frequency at which the interfacial polarization can be generated is likely lower than 10^2^ Hz. In this case, free charges freeze through the relaxation process. As the temperature was increased from −60 to −30 °C, the dielectric response of the interfacial polarization began to appear, especially in a low-frequency range.

At 0 °C, a new plateau with ε′ ~70 could be observed in the frequency range of 10^2^–10^4^ Hz, which was contributed by the interfacial polarization effect. A steplike decrease in the ε′ could be observed in the frequency range of 10^4^–10^5^ Hz. In this case, there existed a decay in polarization with respect to the AC applied electric field [46,47]. The steplike decrease in the ε′ shifted to a high frequency as the temperature was increased. Upon increasing the temperature from 30 to 120 °C, an increase in ε′ could be observed, which was associated with the DC conduction of long-range motion of free charges in the composites.

**Figure 7:**
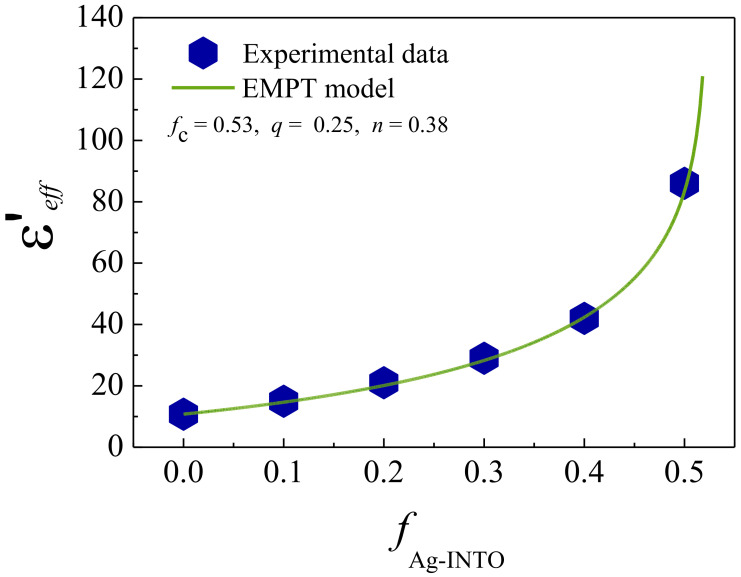
Experimental ε′ values for Ag−INTO/PVDF composites as a function of *f*_Ag__−INTO_ measured at 1 kHz and 20 °C fitted by EMPT model.

Generally, the volume fraction dependence of the ε′ values for the three-phase composites cannot be described by conventional models of the mixing rule such as the effective medium theory and the Maxwell–Garnett, Yamada, and logarithmic models. These models are suitable for two-phase composites without consideration of the interaction between filler and matrix. If interfacial polarization can be induced in the composites, the experimental results cannot be fitted using these models. However, the effective medium percolation theory (EMPT) model can explain the dielectric behavior of three-phase composites containing insulating and conducting fillers. The effective dielectric permittivity (ε′*_eff_*) of three-phase Ag−INTO/PVDF composites was described by considering the following equation:

(1) $$\varepsilon^{\prime }{}_{\mathrm{eff}}=\varepsilon_{\mathrm{PVDF}}\left[1+\frac{f_{n\mathrm{Ag}-\mathrm{INTO}}\left(\varepsilon_{\mathrm{INTO}}-\varepsilon_{\mathrm{PVDF}}\right)}{\varepsilon_{\mathrm{PVDF}}+n\left(1-f_{n\mathrm{Ag}-\mathrm{INTO}}\right)\left(\varepsilon_{\mathrm{INTO}}-\varepsilon_{\mathrm{PVDF}}\right)}\right]{\left|\frac{f_{c}-f}{f}\right|}^{-q},$$

where ε_PVDF_ and ε_INFO_ are the dielectric permittivity of PVDF (~10.8) and INTO (10^4^), respectively, *n* and *q* are the morphology fitting factor and critical exponent, respectively, and *f*_c_ is the percolation threshold. As shown in Figure 7, the best-fitting parameters were *f*_c_ = 0.53, *n* = 0.38, and *q* = 0.25 for the Ag−INTO/PVDF composites. Usually, the theoretical *f*_c_ value of metal/insulator composites is 0.16 (16 vol.%) [2]. According to the fitted result, it is expected that the percolation threshold of this three-phase composite system was higher than the maximum *f*_Ag__−INTO_ value used. It can be confirmed that the percolation network or conduction pathway could not be formed in the composites even when *f*_Ag__−INTO_ = 0.5. The significantly improved dielectric properties of the Ag−INTO/PVDF composites should not be primarily contributed by the percolation effect. This suggestion is consistent with a slightly changed DC conduction (σ_dc_) value, which can be estimated to be a low−frequency σ_ac_ value. According to the percolation theory, *q* ≈ 1.0. The low *q* value indicated a slight change in ε′ near the percolation threshold, which is usually dependent on the conductivity of a filler. The discrete growth of Ag nanoparticles on the surface of the INTO particles prevented the direct contact of Ag nanoparticles, suppressing the increased tanδ. The large value of the *n* parameter indicated the non-spherical shape of the filler particles. The significantly increased ε′ value in the composite with *f*_Ag__−INTO_ = 0.5 was due to the high ε′ of the INTO particles and high intensity of the interfacial polarization at the Ag−INTO and Ag−PVDF interfaces.

## 4. Conclusions

Three-phase polymer matric composites of Ag−INTO/PVDF with different filler volume fractions were fabricated systematically to improve the dielectric properties of a PVDF polymer. The hybrid particles of Ag−INTO were successfully synthesized. Ag nanoparticles were observed to discretely grow on the surface of the INTO particles. The Ag−INTO hybrid particles were dispersed in the PVDF matrix. Notably, the Ag−INTO/PVDF composite with *f*_Ag__−INTO_ = 0.5 exhibited a significantly enhanced ε′ value of ~86 at 1 kHz, while retaining a low tanδ value of ~0.044. Furthermore, ε′ was slightly dependent on the frequency over the measured range (0^2^–10^6^ Hz). Therefore, the incorporation of a PVDF polymer with Ag−INTO hybrid particles is suggested to be an effective method to simultaneously enhance ε′ and reduce tanδ of three-phase composites. The EMPT model was successfully employed to describe the dielectric behavior of the composites. According to the fitted result, it was explained that the enhancement of the ε′ value was caused by the interfacial polarization at the Ag–PVDF and Ag–INTO interfaces and partially resulted from the high ε′ of INTO particles. The low tanδ and conductivity were attributed to the inhibited direct contact between Ag nanoparticles.

## Data Availability

The data presented in this study are available in article.
